# Sex difference in initial thermoregulatory response to dehydrated exercise in the heat

**DOI:** 10.14814/phy2.14947

**Published:** 2021-07-20

**Authors:** Gabrielle E. W. Giersch, Margaret C. Morrissey, Cody R. Butler, Abigail T. Colburn, Zachariah S. Demarais, Stavros A. Kavouras, Ollie Jay, Nisha Charkoudian, Douglas J. Casa

**Affiliations:** ^1^ United States Army Research Institute for Environmental Medicine Natick MA USA; ^2^ Oak Ridge Institute for Science and Education Belcamp MD USA; ^3^ Korey Stringer Institute University of Connecticut Storrs CT USA; ^4^ Hydration Science Laboratory Arizona State University Phoenix AZ USA; ^5^ Thermal Ergonomics Laboratory University of Sydney Sydney NSW Australia

**Keywords:** core temperature, heat production, heat stress, menstrual cycle

## Abstract

Although it is well established that dehydration has a negative impact on thermoregulation during exercise in the heat, it is unclear whether this effect of dehydration is different between men and women, or across the phases of the menstrual cycle (MC). Twelve men and seven women (men: 20 ± 2 years, 70.13 ± 10.5 kg, 173.4 ± 6.0 cm, 54.2 ± 8.6 ml kg^−1^ min^−1^; women: 20 ± 2 years, 57.21 ± 7.58 kg, 161 ± 5 cm, 40.39 ± 3.26 ml kg^−1^ min^−1^) completed trials either euhydrated (urine specific gravity, USG ≤ 1.020, Euhy) or dehydrated (USG > 1.020, Dehy). Trial order was randomized and counterbalanced; men completed two trials (MEuhy and MDehy) and women completed four over two MC phases (late follicular: days 10–13, FDehy, FEuhy; midluteal: days 18–22, LDehy, LEuhy). Each trial consisted of 1.5 h, split into two 30 min blocks of exercise (B1 and B2, 15 min at 11 W/kg & 15 min at 7 W/kg) separated by 15 min rest in between and after. Rectal temperature (*T*_re_) was measured continuously and estimated sweat loss was calculated from the body mass measured before and after each block of exercise. When dehydrated, the rate of rise in *T*_re_ was greater in women in the first block of exercise compared to men, independently of the MC phase (MDehy: 0.03 ± 0.03°C/min, FDehy: 0.06 ± 0.02, LDehy: 0.06 ± 0.02, *p* = 0.03). Estimated sweat loss was lower in all groups in B1 compared to B2 when dehydrated (*p* < 0.05), with no difference between sexes for either hydration condition. These data suggest that women may be more sensitive to the negative thermoregulatory effects of dehydration during the early stages of exercise in the heat.

## Graphical abstract

This study investigated possible sex and menstrual cycle differences to the thermoregulatory response to dehydrated exercise in the heat. We observed a possible augmented core temperature response in women, irrespective of the menstrual cycle phase. Understanding these sex differences will aide in the development of recommendations for all physically active individuals.

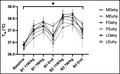

**New and Noteworthy**

Dehydration causes significant decrements in physiological function, including impaired thermoregulation and decreased exercise performance. However, the influences of dehydration in women during exercise in the heat remain poorly understood. We measured thermoregulatory responses to exercise in a hot, humid environment in men and in women during two phases of the MC. We observed an increased rate of rise in core temperature at the beginning of the exercise in the heat in women when dehydrated, which was unaffected by the MC. Our results suggest that during mild dehydration, there is greater heat storage at the onset of exercise in women compared to men.

## 1 INTRODUCTION

Dehydration is detrimental to thermoregulatory function during exercise in the heat, resulting in an inhibition of the reflex heat dissipation mechanisms of sweating and cutaneous vasodilation, and more rapid increases in the core body temperature (Fortney et al., 1984; Sawka et al., 1989). Mechanisms of thermoregulation are also altered by menstrual cycle (MC) hormones and with other changes in reproductive hormone status, such as oral contraceptive use and post‐menopausal hormone replacement therapy (Brooks‐Asplund et al., 2000; Charkoudian & Johnson, 1997, 2000). However, it is currently unclear whether the influences of dehydration and the influences of reproductive hormone status (or biological sex) have any interaction with each other in terms of a net effect on thermoregulation during exercise in the heat. This is valuable data to inform recommendations for health and safety for men and women during hydration and heat stress.

Thermoregulatory differences between men and women during exercise in the heat in a euhydrated state are often attributed to anthropometric and/or morphological differences (Gagnon & Kenny, 2012; Jay, 2014; Shapiro et al., 1980). Previously, body size has been shown to account for differences in thermoregulatory responses when exercise intensity is determined based on relative maximal oxygen uptake (VO_2max_; Gagnon et al., 2009). This has been addressed by matching absolute metabolic heat production relative to body mass (kg) or body surface area (m^2^) which is considered to be the ideal approach for assessing thermoregulatory responses to exercise and allows for unbiased comparisons between sexes (Cramer & Jay, 2014; Gagnon et al., 2008). However, in studies where exercise intensity was prescribed based on heat production (Gagnon et al., 2008, 2009; Gagnon & Kenny, 2012), women have primarily been investigated in the early follicular phase of the MC when sex hormone concentrations are at their lowest point of the cycle (Owen, 1975). This has minimized the ability to evaluate potential influences of estradiol and/or progesterone in contributing to differences between the sexes.

Female sex hormones fluctuate significantly throughout the human MC and have a quantitative impact on thermoregulatory function (Charkoudian & Stachenfeld, 2014, 2016; Kolka & Stephenson, 1989, 1997a, 1997b; Owen, 1975). Women have an increased *T*_re_ at rest, during exercise, and post‐exercise in the midluteal phase compared to the early follicular phase (Carpenter & Nunneley, 1988; Giersch, Morrissey, et al., 2020; Kolka & Stephenson, 1997b; Kuwahara et al., 2005; Lei et al., 2017). The increased *T*_re_ is likely due to higher progesterone concentrations, which increases the regulated *T*_re_ set‐point and subsequent onset for heat dissipation mechanisms (Charkoudian & Stachenfeld, 2016; Kolka & Stephenson, 1997b). While hormonal changes alter absolute temperature and threshold for thermoeffectors’ function, without influence on the overall change in core temperature (Kolka & Stephenson, 1997b), suggesting that menstrual phase would impact absolute temperature but not change or rate of rise. However, these hormonal influences may result in differential responses in women compared to men during similar heat stress; however, these questions have not been addressed with respect to varying hydration status (Sawka et al., 1998).

Additionally, a majority of the research conducted to date has investigated thermoregulatory differences in euhydrated conditions. It is well understood that dehydration impairs thermoregulation during intense exercise in the heat and is considered a risk factor for developing exertional heat illnesses (Casa et al., 2015; Cleary, 2007; Gardner et al., 1996). Dehydration compromises thermoregulation during exercise in the heat by impairing heat dissipation capacity by compromising both sweating and convective transfer of heat from the core to the periphery via blood flow (Graham et al., 2020; Sawka, 1992; Sawka et al., 1998). Sex, reproductive hormones, and hydration status may alter thermoregulatory responses to exercise in the heat, but the mechanisms involved remain incompletely understood. Therefore, the purpose of this investigation was to assess whether the influence of sex on thermoregulatory responses to exercise in the heat are independently modified by hydration status, and further altered by the MC phase in young healthy men and women. We hypothesize that greater rise in the core temperature during exercise with dehydration will be exacerbated in women independently of the MC phase.

## 2 METHODOLOGY

### 2.1 Ethical approval

All study protocols and procedures were conducted in accordance with the Declaration of Helsinki and were evaluated and approved by the Institutional Review Board at the University of Connecticut (Protocol Nos. H18‐220 and H19‐049).

### 2.2 Subjects

A total of 23 participants, 12 men and 11 women, were recruited to participate in this investigation. All participants were screened for contraindications to exercise in the heat and were excluded prior to enrollment if they had history of past heat illness within 3 years, chronic health issues affecting the endocrine or thermoregulatory systems, or had used hormonal contraceptives within the previous 6 months. Subject characteristics are shown in Table 1. Three women were excluded from these analyses due to their inability to complete exercise at the prescribed intensities due to calculations for 11 W/kg heat production yielding speeds >120% their peak velocity during maximal oxygen uptake testing (VVO_2max_). One additional woman completed all components of the protocol and was removed post hoc based on sex hormone concentration outside the expected range and two standard deviations from the mean for each phase (i.e., estradiol > 1,100 pg/ml and in both MC phases). Therefore, data for women are reported for *n* = 7. Power analyses were conducted on the data post hoc and *n* = 7 was found to provide sufficient statistical power for included comparisons.

**TABLE 1:** Subject characteristics. *denotes *p* < 0.05 for comparison between men and women. Data are presented as mean ± *SD*. BSA represents the body surface area

	Age (year)	Weight (kg)	Height (cm)	BSA (m^2^)	BSA:mass (m^2^/kg)	Body fat (%)
Men *n* = 12	20 ± 2	70.13 ± 10.5*	173 ± 6*	1.83 ± 0.15*	0.026 ± 0.001*	10 ± 2*
Women *n* = 7	20 ± 2	57.21 ± 7.58	161 ± 5	1.60 ± 0.13	0.028 ± 0.002	14 ± 4
Total *n* = 19	20 ± 2	61.91 ± 10.60	167 ± 7	1.69 ± 0.17	0.027 ± 0.002	13 ± 4

Body surface area (BSA) was estimated (Du Bois, 1916) from height measured via a wall‐mounted stadiometer (Seca, model 220) and weight (Ohaus Defender 3000, Ohaus Corporation). Participants underwent a test of maximal oxygen uptake (VO_2max_) prior to participation in the study in order to ensure that fitness criteria were met (men: VO_2max_ > 45 ml kg^−1^ min^−1^, women: VO_2max_ > 35 ml kg^−1^ min^−1^). Fitness criteria were chosen in an attempt to best ensure the ability of participants to complete exercise trials at prescribed intensities. Participants ran on a treadmill in 3‐min stages until volitional exhaustion. Expired air was collected and analyzed using indirect calorimetry (TrueOne 2400, ParvoMedics) in order to calculate VO_2max_ (ml kg^−1^ min^−1^). Following VO_2max_ testing, participants underwent 7‐site skinfold assessment to determine body composition from standardized guidelines (Medicine ACoS, 2013).

In total, men completed two exercise trials (described below), one beginning in a euhydrated state (urine specific gravity, USG ≤ 1.020) and one beginning in a dehydrated state (USG > 1.020; MEuhy and MDehy, respectively). These data were a part of a larger investigation in which the fluid regulatory data have been previously published (Giersch, Colburn, et al., 2020). Women completed four trials, one starting euhydrated and dehydrated in the follicular (F, days 10–13) and the luteal (L, days 18–22) phases of the MC (FDehy, FEuhy, LDehy, and LEuhy, respectively).

### 2.3 Heat production calculation

To calculate heat production, participants underwent a test at familiarization where they exercised for 24 min in 4‐min stages at 30%, 40%, 50%, 60%, 70%, and 80% velocity of their VO_2max_, respectively, and 2% grade on the treadmill to account for terrain and wind resistance. Environmental conditions were equivalent to exercise trials (~33°C, 50% relative humidity) to allow for the appropriate estimation of heat production during exercise trials. Expired air was collected and analyzed for VO_2_ (L/min), and respiratory exchange ratio via indirect calorimetry (TrueOne 2400, ParvoMedics). These variables were used in order to calculate metabolic heat production utilizing previously described methods (Cramer & Jay, 2019). Two exercise intensities, based on prescribed heat production, were utilized in this trial, a higher intensity at 11 W/kg and a lower intensity at 7 W/kg. Exercise intensities were chosen to elicit a running or jogging intensity (11 W/kg) and a lower intensity walk (7 W/kg) these intensities were chosen through protocol development that best elicited differing responses. Exercise intensities for included participants are shown in Table 2.

**TABLE 2:** Exercise intensities for prescribed heat production between men and women. *denotes *p* < 0.05 within the variable. Data are presented as mean ± *SD*

	Men (*n* = 12)	Women (*n* = 7)
VO_2max_	54.2 ± 8.6*	40.4 ± 3.3
V_VO2max_ (mph)	8.4 ± 1	7.0 ± 0.6
Velocity at 11 W/kg (mph)	4.9 ± 1.0	5.5 ± 1.1*
Relative VO_2_ at 11 W/kg (% VO_2max_)	60.4 ± 15.8	80.4 ± 16.2*
Velocity at 7 W/kg (mph)	2.7 ± 0.6	3.2 ± 0.8*
Relative VO_2_ at 7 W/kg (%VO_2max_)	33.7 ± 10.1	46.7 ± 11.9*

### 2.4 Protocol

Pre‐exercise dehydration was induced via 24‐h fluid restriction immediately prior to the exercise trial and confirmed via USG > 1.020. Participants were instructed to consume no fluid during the 24‐h pre‐exercise period and to consume foods with lower fluid content (i.e., avoid apples, grapes, soup, etc.). Twenty‐four‐hour fluid restriction was chosen as the pre‐exercise dehydration stimulus because it provides a practically relevant low level of dehydration, similar to that of someone who is a low fluid consumer or an individual who does not adequately recover from dehydrating exercise the day before (Cheuvront & Kenefick, 2014). Urine pregnancy tests were conducted immediately prior to exercise trials for women (Sure‐Vue STAT Serum/Urine hCG Test Kit, Thermo Fisher Scientific). Following venous cannula insertion, participants were permitted to consume 200 ml of water prior to entering the environmental chamber but no fluid consumption was permitted throughout the course of the exercise trial. All participants started each exercise bout in the morning, between 06:00 and 11:00, and participants performed subsequent trials within one hour of previous trial start times to reduce the possible impact of diurnal variation on internal temperature. For men and women, trials were conducted at least 72 h apart to reduce the influence of hydration conditions between trials, and over the course of 2–3 MCs in women based on randomization and trial order.

**FIGURE 1:**
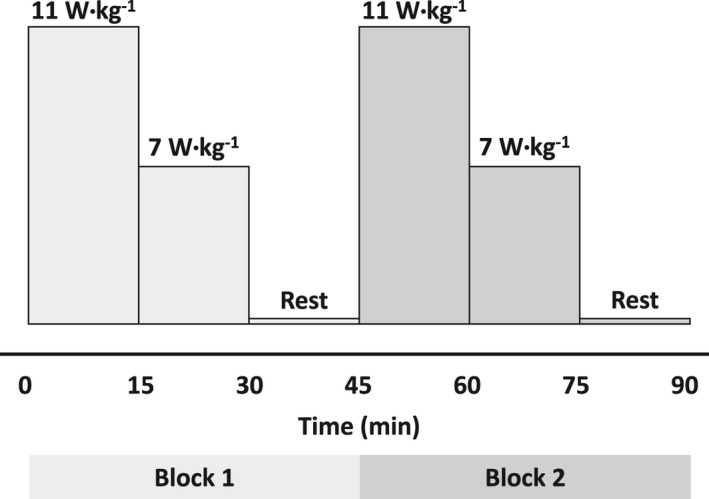
Schematic representation of the exercise protocol. Exercise intensity is represented by 11 and 7 W/kg of heat production. Note, the third block of exercise was included in the trials, but was not included in the analyses due to the lack of standardization, as described in the methods

All exercise heat tests were conducted in an environmental chamber (CANTROL, Environmental Systems Limited) at 33.8 ± 1.3°C, 49.5 ± 4.3% relative humidity. Exercise trials consisted of two identical 45‐min blocks of exercise (B1, B2), which included 11 W/kg of heat production for 15 minutes, 7 W/kg of heat production for 15 min, and seated resting in the chamber for 15 min. A schematic representation of the exercise protocol is shown in Figure 1. The original design for this study included a third identical block, however, we could not include those data for analysis due to the inability of many volunteers (*n* = 9) to maintain the prescribed exercise intensity during this block. Thus, an inability to standardize the protocol across participants led to the exclusion of the third block from the analysis of variables collected during exercise (e.g., *T*_re_, sweat rate).

Whole‐body sweating rate and body mass loss were calculated from nude body mass pre‐ and immediately post‐trial (following three blocks of exercise—third block not included in this analysis) on a scale (Ohaus Defender 3000, Ohaus Corporation), and corrected for urinary loss. Body mass change during exercise was estimated from clothed body mass following each block during the exercise protocol. Estimated sweat losses during the exercise blocks were calculated from minimally clothed body mass (participants without their shoes) measured pre‐exercise and following each block of exercise. Estimated sweat loss relative to BSA (m^2^) and mass (kg) was also calculated via total estimated sweat loss divided by individual BSA measures, calculated using previously described methods (Du Bois, 1916), and nude body mass. Internal body temperature (*T*_re_) via rectal thermistor (400 series, YSI 400 Incorporated) and heart rate from a 3‐lead electrocardiogram (ECG100C, BIOPAC Systems Incorporated) were collected continuously throughout the exercise trial utilizing a physiological monitoring system (MP160, BIOPAC Systems Incorporated). Change in *T*_re_ (Δ*T*_re_) was calculated for each intensity level throughout each block of exercise. The rate of rise in *T*_re_ was calculated from Δ*T*_re_ divided by the time that participants exercised at each level of fixed heat production (15 min).

### 2.5 Hormonal analysis

Trials within MC phases were scheduled utilizing self‐reported menstrual history questionnaires and verified post hoc utilizing estrogen and progesterone enzyme‐linked immunoassays (ALPCO Diagnostics). Female sex hormone concentrations are shown in Table 3. Women were tested over the course of two to three MCs based on a priori trial order randomization.

**TABLE 3:** Sex hormone concentration. Data are presented as mean ± *SD*. *denotes *p* < 0.05 between both follicular phase trials

	Follicular dehydrated	Follicular euhydrated	Luteal dehydrated	Luteal euhydrated
Estradiol (pg/ml)	112.33 ± 14.70	122.40 ± 16.56	130.82 ± 24	121.48 ± 24.48
Progesterone (ng/ml)	1.16 ± 0.31	0.92 ± 0.18	5.19 ± 1.56*	4.50 ± 1.38*

### 2.6 Statistical analysis

After confirming data normality, a univariate analysis of variance (ANOVA) was used to determine the effect of time and hydration status on *T*_re_ with sex and MC included as independent variables. Two three‐way ANOVAs were conducted to assess possible interactions between time, hydration status, and sex. Additionally, one‐way ANOVAs were conducted in order to assess differences in thermoregulatory variables for between‐subject factors including sex, utilizing a Bonferroni post hoc analysis. A two‐by‐two repeated‐measures ANOVA was conducted to assess within‐subject factors including MC phase and hydration conditions in women with Bonferroni pairwise comparisons. All statistical analyses were conducted utilizing SPSS software (v27 IBM Corporation).

## 3 RESULTS

**FIGURE 2:**
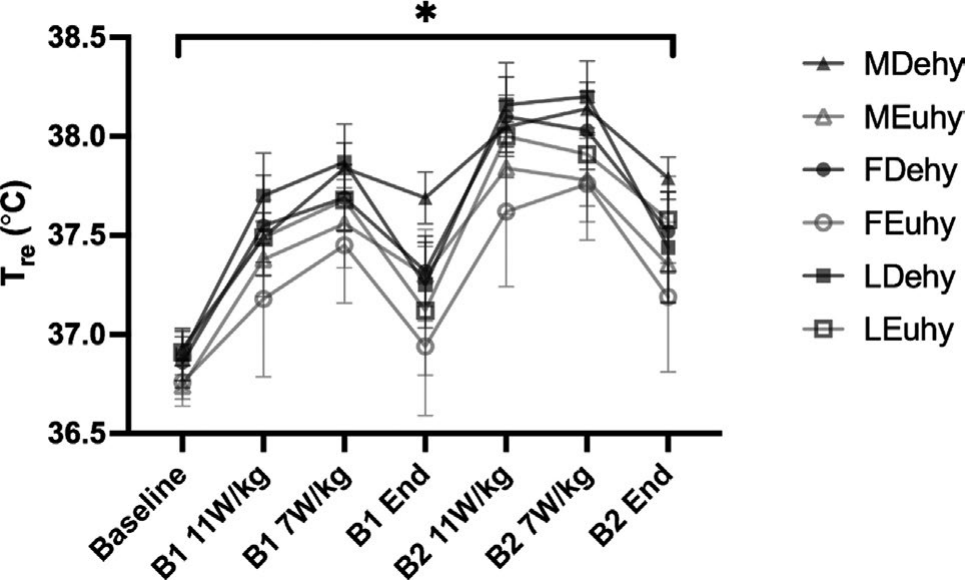
Absolute *T*_re_ measures collected at the end of each exercise intensity within each block analyzed via three‐way analysis of variance, *n* = 12 men, *n* = 7 women. B1 = block 1, B2 = block 2. 11 and 7 W/kg are representative of prescribed heat production intensities. *denotes the significant main effect of time and hydration status across groups (*p* < 0.001 for both variables). Data are presented as mean ± *SE*

Twenty‐four‐hour fluid restriction yielded similar body mass loss across groups (~1%) with no influence of sex or MC phase (men: −1.18 ± 0.91%, follicular: −1.15 ± 0.85%, luteal: −0.74 ± 0.41%, *p* > 0.05). Men had greater body mass and BSA, but lower BSA to mass ratio (BSA:mass) than women, as shown in Table 1. No differences in baseline *T*_re_ were observed between men and women, MC phases, or hydration conditions (MDehy: 36.93 ± 0.10°C, MEuhy: 36.74 ± 0.09°C, FDehy: 36.87 ± 0.10°C, FEuhy: 36.82 ± 0.10°C, LDehy: 37.02 ± 0.13°C, LEuhy: 36.94 ± 0.08°C, *p* = 0.843, *p* = 0.356, *p* = 0.774, respectively). Both time and hydration status had a significant effect on *T*_re_, such that *T*_re_ increased as exercise continued, and *T*_re_ was higher for groups when dehydrated (*p* < 0.001) as shown in Figure 2. Heart rate increased as expected in each block of exercise but did not appear to be affected by hydration conditions (data not shown).

No differences between sexes or MC phases were observed in absolute *T*_re_ (°C) throughout the exercise trial (Figure 2). Table 4 shows all Δ*T*_re_ and *T*_re_ rate of rise data. In Dehy trials, men showed a smaller rate of rise in *T*_re_ in B1 11 W/kg compared to women (MDehy: 0.03 ± 0.03°C/min vs. FDehy: 0.07 ± 0.02°C/min, LDehy: 0.06 ± 0.02°C/min; *p* = 0.01, 0.03, respectively). In Dehy B2, men also had a significantly lower rate of rise compared to women in the luteal phase (MDehy: 0.02 ± 0.02°C/min, LDehy 0.06 ± 0.03°C/min, *p* = 0.002). In Dehy, men experienced significantly lower Δ*T*_re_ in B2 compared to women in the luteal phase (MDehy: 0.36 ± 0.23°C, LDehy: 0.92 ± 0.46°C, *p* < 0.01). Comparing blocks, women in the follicular phase in Dehy exhibited significantly greater Δ*T*_re_ in B1 compared to B2 (FDehy B1: 0.78 ± 0.14°C, FDehy B2: 0.57 ± 0.10°C, *p* = 0.042) as well as the rate of rise (FDehy B1: 0.07 ± 0.02°C/min, FDehy B2: 0.03 ± 0.01°C/min, *p* = 0.025). We observed no differences in Δ*T*_re_ in men between Euhy and Dehy trials, possibly due to the relatively mild nature of the dehydration, with all dehydrated groups beginning exercise ~1% body mass loss.

**TABLE 4:** *T*_re_ rate of rise and Δ*T*_re_ data for all groups. Comparisons conducted via one‐way analysis of variance with post hoc comparisons (sex differences) and repeated‐measures analysis of variance with pairwise comparisons (menstrual cycle differences) for *n* = 12 men, and *n* = 7 women. *denotes differences (*p* < 0.05) compared to men in the same hydration condition, ^^^denotes differences (*p* < 0.05) between hydration conditions in the same group, and ^#^denotes differences (*p *< 0.05) between blocks

	*T*_re_ rate of rise (°C/min)		Δ*T*_re_ (°C)			
	B1	B2	B1		B2	
			11 W/kg	7 W/kg	11 W/kg	7 W/kg
MDehy	0.03 ± 0.03	0.02 ± 0.02	0.61 ± 0.41	0.28 ± 0.20	0.36 ± 0.23	0.08 ± 0.11
MEuhy	0.04 ± 0.03	0.03 ± 0.02	0.76 ± 0.39	0.11 ± 0.24	0.53 ± 0.31	−0.08 ± 0.18^^^
FDehy	0.07 ± 0.02*	0.04 ± 0.02^#^	1.02 ± 0.29	0.15 ± 0.26	0.69 ± 0.21	−0.16 ± 0.27
FEuhy	0.05 ± 0.04	0.05 ± 0.01	0.77 ± 0.58	0.25 ± 0.35	0.74 ± 0.21	0.08 ± 0.41
LDehy	0.06 ± 0.02*	0.06 ± 0.03*	0.94 ± 0.26	0.20 ± 0.25	0.92 ± 0.46*	0.06 ± 0.20
LEuhy	0.05 ± 0.04	0.04 ± 0.02	0.74 ± 0.64	0.05 ± 0.28	0.68 ± 0.23	0.12 ± 0.34

**FIGURE 3:**
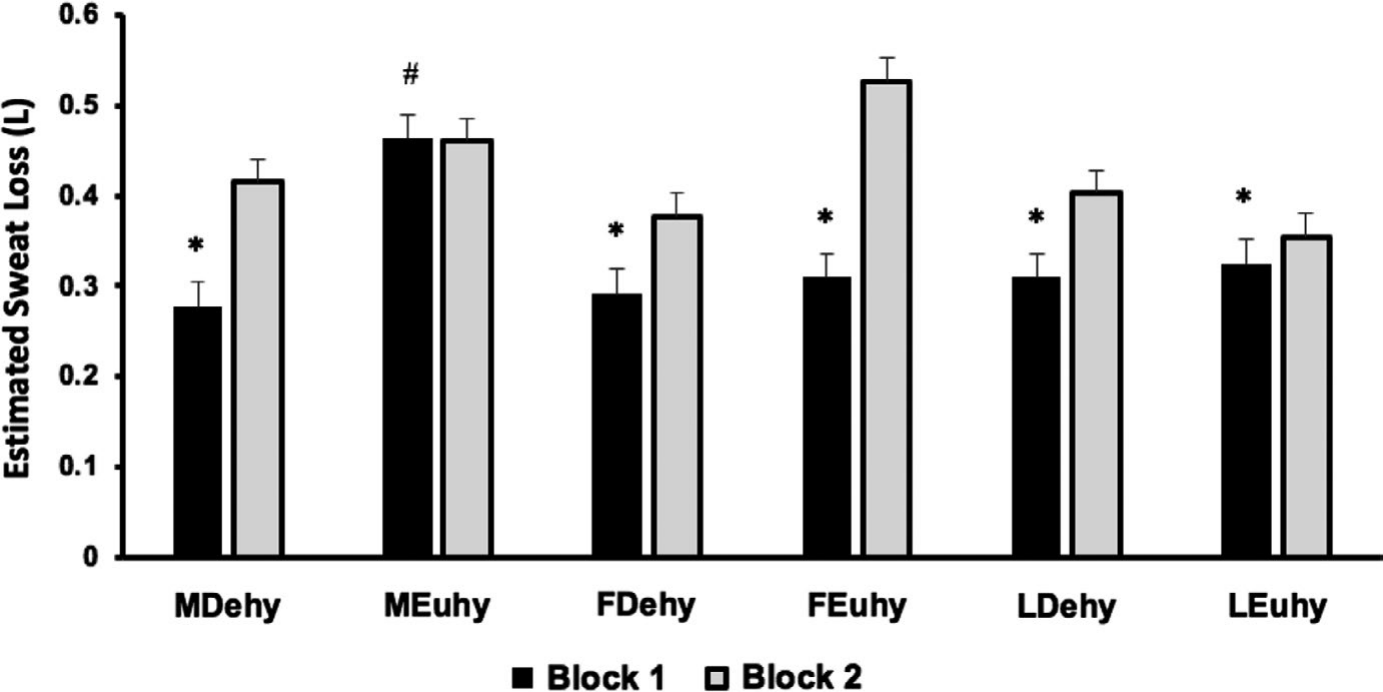
Total estimated sweat loss in each exercise block by trial analyzed via analysis of variance with post hoc comparisons (sex differences) and repeated‐measures analysis of variance with pairwise comparisons (menstrual cycle differences), *n* = 12 men, *n* = 7 women. MEuhy > MDehy, B1 < B2 across all trials/phase except men Euhy. *denotes *p* < 0.05 compared to B2 within the trial. ^#^denotes *p* < 0.05 between MDehy and MEuhy. Data are presented as mean ± *SE*

During exercise, men had significantly lower estimated sweat loss in B1 compared to B2 when in MDehy but did not exhibit any differences in MEuhy as shown in Figure 3. Conversely, women experienced lower estimated sweat loss in B1 (vs. B2) across hydration status and MC phase (Figure 2). No sex or MC differences in the Euhy trials were observed in total estimated sweat loss. Estimated sweat loss relative to BSA and mass showed similar differences within groups with B1 less than B2 in MDehy, and women across all trials without any observed effect of sex or MC phase. Exercise heat stress elicited further dehydration with no impact of sex or MC phase (MDehy: −1.90 ± 0.51%, MEuhy: −2.17 ± 0.41%, FDehy: −1.76 ± 0.64%, FEuhy: −2.42 ± 1.22%, LDehy: −2.26 ± 0.59%, LEuhy: −2.28 ± 0.54%, *p* > 0.05).

## 4 DISCUSSION

To our knowledge, this is the first investigation to assess differences in thermoregulation between sexes, MC phases, and hydration states at two different intensities of exercise while controlling for heat production (i.e., W/kg). The major finding of the present study is that initial hydration status exacerbates the rise in *T*_re_ at the beginning of the exercise in the heat in women as compared to men, even at the relatively mild level of dehydration observed. Our results suggest that women have an augmented response compared to men at the beginning of the exercise in the heat, indicated by the increased rate of rise across both phases. The second block of exercise (B2) was also associated with an increased rate of rise and Δ*T*_re_ in women compared to men. These differences in *T*_re_ were present without any differences in estimated sweat loss (absolute or relative to BSA). This would suggest that observed differences in sex were not related to evaporative heat loss. Another possible mechanism suggested by the recent work of Graham et al (Graham et al., 2020) is a change in the internal distribution of heat from the core to the periphery. This internal distribution, via convective transfer (i.e., blood flow), is decreased in dehydrated individuals; this dehydration‐mediated shift may have been augmented in our present female volunteers during the blocks in which delta Tre was higher.

As is typically observed, the men in our study had higher BSA but lower BSA: mass ratios compared to women. The larger BSA: mass ratio in women may have contributed to the differences observed in Δ*T*_re_ and rate of rise in *T*_re_ we observed in this uncompensable environment (i.e., increased heat gain from the environment). However, recent work from Ravanelli and colleagues in large and small men argues against this possibility, since both groups had similar increases in core temperature in an uncompensable environment similar to that of the present study (Ravanelli et al., 2017). As noted above, any possible disadvantage would have been augmented by dehydration and the distribution of heat in the body via blood flow (Graham et al., 2020), since skin blood flow responses tend to be lower in dehydrated individuals for a given hyperthermic stimulus (Sawka et al., 1998). Blood flow distribution itself may be altered based on the MC phase or circulating reproductive hormones (Charkoudian & Johnson, 1997, 1999a, 1999b). Since the core temperature was assessed rectally in this study, it is possible that the concentration of blood flow around the thermistor was affected by blood distribution as has been previously discussed (Graham et al., 2020) and observations may have been different if esophageal thermistors had been used.

Another possible mechanism that may contribute to thermoregulatory differences between men and women is differences in body composition. Anthropometric characteristics have been previously concluded to be the primary factor for observed thermoregulatory differences between sexes (Gagnon et al., 2009). In the present investigation, body composition was assessed utilizing skinfolds that rely on the assessment of subcutaneous fat, which is the primary driver for the insulative factors associated with thermal balance. However, given the relatively small differences in body fat between men and women (~10% vs. ~14%), and the fact that much larger differences in adiposity did not appear to make large differences in heat storage (Dervis et al., 2016; Jay et al., 2011), we believe it is unlikely that the body fat content was a large contributor to our present findings.

While we were not able to directly assess the *T*_re_ thresholds for sweating in our study, we speculate that our results may suggest a sex difference in the influence of hydration on this threshold. Men experienced significantly greater estimated sweat loss in Euhy B1 compared to Dehy B1, where women observed no differences in estimated sweat loss between hydration conditions. This is consistent with previous research showing a threshold shift in the onset of sweating with dehydration in men (Sawka et al., 1989). Women experienced lower estimated sweat loss in B1 compared to B2 in both phases of the MC regardless of hydration condition, and there was no effect of hydration status on sweat losses in women in either block. To our knowledge, the previously observed threshold shift in sweating with dehydration in men (Sawka et al., 1989), has not been studied or replicated in women, and warrants future investigation.

The MC phase did not appear to have an effect on any thermoregulatory variables measured in this investigation. We were surprised to note that we did not observe a higher baseline temperature in the luteal phase when compared to the follicular phase, based on the repeated observation that this is usually the case (Kolka & Stephenson, 1997b). Differences in menstrual phase resulted in a non‐significant ~0.2°C increase in internal body temperature in the luteal phase. Even though this difference was not statistically significant, it was just lower than 0.3–0.5°C that has been consistently previously reported (Carpenter & Nunneley, 1988; Kolka & Stephenson, 1997a, 1997b; Kuwahara et al., 2005). This increase in *T*_re_ is thought to be caused by progesterone (Kolka & Stephenson, 1997b), but our data show no significant relationship between progesterone and any measure of *T*_re_ despite the significant increase in progesterone concentration shown in the luteal phase. While progesterone concentration was higher in the luteal phase, it was not elevated to the degree that is expected from the midluteal phase in a typical ovulatory cycle (Speroff & Wiele, 1971). This could suggest the progesterone peak was missed in the scheduling of exercise trials and possibly, that the thermoregulatory effect of progesterone may be dose‐dependent (Baker et al., 2020).

From a big picture perspective, current governing body recommendations have been developed based almost exclusively on data collected in men (Armstrong et al., 2007; Casa et al., 2015). Incorporating data on women and sex differences is important for future recommendations to maintain health and safety during exercise heat stress. While more research is warranted, our present data contribute novel information on the possible effects of dehydration on initial thermoregulatory responses between men and women. Specifically, despite the relatively small numerical differences in Δ*T*_re_ and rate of rise in *T*_re_, these findings suggest a sex difference in thermoregulation when dehydration is present, prompting a possible need for sex‐specific recommendations in exercise heat stress. Future studies should continue to investigate sex and hormonal differences with respect to additional factors that may affect thermoregulation during exercise in the heat and incorporate higher levels of dehydration in order to gain a comprehensive understanding of any differences.

## 5 LIMITATIONS

The small sample size in women is the primary limitation of this investigation. Due to the matching of heat production, three women had to be excluded from this original data set based on unachievable exercise intensities, thus it is possible that some comparisons may be underpowered to assess significant differences based on sex. Additionally, the heat production visit was conducted only in the early follicular phase of the MC, where heat production may differ based on the running economy (Smoljanic et al., 2014).

## 6 CONCLUSIONS

We report here that dehydrated women had an augmented rate of rise in T_re_ at the beginning of the exercise in the heat when compared to dehydrated men. We observed no differences in thermoregulatory responses based on hormonal status. Hydration status did appear to impact sweating rate in both men and women, suggesting that dehydration does impair thermoregulatory response in women, as has been previously observed in men. Future research should seek to include women, and hormonal comparisons to assess possible variation in thermoregulatory mechanisms, including in the thresholds for cutaneous vasodilation and sweating, as potentially influenced by hydration status. Our present results emphasize the importance of continuing to investigate responses specific to women, especially since results such as these may inform future recommendations for exercise in the heat for military, athletic and other populations who are likely to be physically active in hot environments.

## CONFLICTS OF INTEREST

S.A.K. has provided scientific consultation to Danone Research and Quest Diagnostics and has active research grants with Danone Research and Standard Process. D.J.C. has served as an expert witness and received consulting honoraria from Clif Bar, Sports Innovation Laboratories, and the National Football League, funding from Gatorade, and royalties from Jones and Bartlett, Springer, LWW, Wolters‐Kluwer Publishers, Up‐to‐Date, and Routledge/ Taylor & Francis Group. G.E.W.G, M.C.M, C.R.B., A.T.C., Z.S.D, O.J., and N.C. have no conflicts, financial or otherwise, to disclose.

## AUTHOR CONTRIBUTIONS

G.E.W.G, O.J., and N.C. conceived and designed the research; G.E.G., M.C.M., C.R.B., and Z.S.D. performed the experiments; G.E.W.G. and A.T.C. analyzed the data; G.E.G., A.T.C., S.A.K., O.J., and N.C. interpreted the results of experiments; G.E.G. prepared tables and figures; G.E.W.G. drafted the manuscript; G.E.W.G., A.T.C., M.C.M., C.R.B., Z.S.D., S.A. K., O.J., N.C., and D.J.C. edited and revised the manuscript; G.E.G., A.T.C., M.C.M., C.R.B., Z.A.S., S.A.K., O.J., N.C., and D.J.C. approved the final version of the manuscript.

## DISCLAIMER

The views, opinions, and/or findings contained in this article are those of the authors and should not be construed as an official United States Department of the Army position, or decision, unless so designated by other official documentation. Approved for public release; distribution unlimited. Citations of commercial organizations and trade names in this report do not constitute an official Department of the Army endorsement or approval of the products or services of these organizations.
